# *Pseudocochlodinium profundisulcus* Resting Cysts Detected in the Ballast Tank Sediment of Ships Arriving in the Ports of China and North America and the Implications in the Species’ Geographic Distribution and Possible Invasion

**DOI:** 10.3390/ijerph19010299

**Published:** 2021-12-28

**Authors:** Lixia Shang, Xinyu Zhai, Wen Tian, Yuyang Liu, Yangchun Han, Yunyan Deng, Zhangxi Hu, Ying Zhong Tang

**Affiliations:** 1 CAS Key Laboratory of Marine Ecology and Environmental Sciences, Institute of Oceanology, Chinese Academy of Sciences, Qingdao 266071, China; lxshang@qdio.ac.cn (L.S.); zhaixinyu16@mails.ucas.edu.cn (X.Z.); liuyuyang@qdio.ac.cn (Y.L.); yunyandeng@qdio.ac.cn (Y.D.); 2 Laboratory for Marine Ecology and Environmental Science, Qingdao National Laboratory for Marine Science and Technology, Qingdao 266237, China; 3 Center for Ocean Mega-Science, Chinese Academy of Sciences, Qingdao 266071, China; 4 State Key Laboratory of Ballast Water Research, Comprehensive Technical Service Center of Jiangyin Customs, Wuxi 214440, China; tianwen@jytc.org.cn (W.T.); hanyangchun@jytc.org.cn (Y.H.)

**Keywords:** biological invasion, dinoflagellate, harmful algal blooms (HABs), *Pseudocochlodinium profundisulcus*, resting cyst, ships’ ballast tank sediment

## Abstract

Over the past several decades, much attention has been focused on the dispersal of aquatic nonindigenous species via ballast tanks of shipping vessels worldwide. The recently reclassified dinoflagellate *Pseudocochlodinium profundisulcus* (previously identified as *Cochlodinium* sp., *Cochlodinium geminatum*, or *Polykrikos geminatus*) was not reported in China until 2006. However, algal blooming events caused by this organism have been reported almost every year since then in the Pearl River Estuary and its adjacent areas in China. Whether *P. profundisulcus* is an indigenous or an invasive species has thus become an ecological question of great scientific and practical significance. In this study, we collected the sediments from ballast tanks of ships arriving in the ports of China and North America and characterized dinoflagellate resting cysts via a combined approach. We germinated two dark brownish cysts from the tank of an international ship (Vessel A) arriving at the Jiangyin Port (China) into vegetative cells and identified them as *P. profundisulcus* by light and scanning electron microscopy and phylogenetic analyses for partial LSU rDNA sequences. We also identified *P. profundisulcus* cyst from the ballast tank sediment of a ship (Vessel B) arriving in the port of North America via single-cyst PCR and cloning sequencing, which indicated that this species could be transported as resting cyst via ship. Since phylogenetic analyses based on partial LSU rDNA sequences could not differentiate all sequences among our cysts from those deposited in the NCBI database into sub-groups, all populations from China, Australia, Japan, and the original sources from which the cysts in the two vessels arrived in China and North America were carried over appeared to share a very recent common ancestor, and the species may have experienced a worldwide expansion recently. These results indicate that *P. profundisulcus* cysts may have been extensively transferred to many regions of the world via ships’ ballast tank sediments. While our work provides an exemplary case for both the feasibility and complexity (in tracking the source) of the bio-invasion risk via the transport of live resting cysts by ship’s ballast tanks, it also points out an orientation for future investigation.

## 1. Introduction

A ship’s ballast tanks carrying ballast water and sediments were proved to be responsible for the dispersal of toxic phytoplankton worldwide [1,2,3,4,5,6,7]. Recently, a large number of studies have focused on the introduction potency of ballast sediments, which can be accumulated in the bottom of the tanks and are difficult to be washed off [8,9,10]. For instance, tens to hundreds of tons of fine mud with a mean particle size of <20 μm were found in tanks, with the thickness ranging from several centimeters to >30 cm [11]. The ballast sediments provide a suitable environment for dinoflagellates resting cysts, which could remain and maintain viable in sediments for years [12,13,14,15,16]. Hence, there were usually more dinoflagellate cells (cysts) within ballast sediments compared to that in ballast water, e.g., >300 million *Alexandrium* cysts existing in sediments of one single ballast tank in a ship entering an Australian port [1], which implied the great threat of ballast tank sediments to marine ecosystem, human health, and economic development [1,10,17,18].

Gymnodiniales are the major order of dinoflagellates, many species of which have ecological significance due to the ability to form harmful algal blooms (HABs). Among them, *Pseudocochlodinium profundisulcus* (reported as *Cochlodinium* sp., *Cochlodinium geminatum* or *Polykrikos geminatus* [19]) was firstly reported to form bloom in April 2006 in the coastal water of Zhuhai, Guangdong province, China [20]. Since then, blooms caused by this species have occurred frequently in the South China Sea, whereas blooming areas from several to 300 km^2^ have the maximum cell density up to 4.13 × 10^7^ cells·L^−1^ [21,22,23,24,25]. These blooms not only caused severe water discoloration but also led to economic loss, for instance, a small-scale bloom (89 km^2^) in August 2011 caused a loss of about 3.16 million RMB [24]. Moreover, *P. profundisulcus* bloom water exhibited a toxic effect on zooplankton *Artemia salina* [26]. Recently, Dong et al. (2020) demonstrated that retinal (a potential teratogenic agent) existed in cultured *P. profundisulcus* and also accumulated rapidly during the *P. profundisulcus* bloom, implying its potential risk to aquatic organisms [23]. Furthermore, *P. profundisulcus* culture significantly inhibited the growth of co-occurring phytoplankton, including *Prorocentrum micans*, *Heterosigma akashiwo*, *Karlodinum veneficum,* and *Rhodomonas salina*, which suggests that the allelopathy of *P. profundisulcus* may play an important role in competing with other phytoplankton and staying dominant throughout blooms [27].

*Pseudocochlodinium profundisulcus* has been reported in coastal waters of China, Japan, Australia, and other regions [19,28,29,30]. Lan and Gu (2014) reported that *P. profundisulcus* could produce resting cyst in the Pearl River estuary, implying that cyst germination may provide a source for blooms [28]. Recently, our group observed that the resting cysts of *P. profundisulcus* in some ship’s ballast sediment samples [10], which suggests this important HABs-causing species in China might be associated with bio-invasion risk. However, whether *P. profundisulcus* is a local population or an invasive species has become a question of great scientific and practical significance, which was urgent to be studied to understand the mechanism of bloom explosive and transmission better.

In this study, we used a combinatorial approach including morphological observation, resting cyst germination, molecular identification, and phylogenetic analyses to (1) investigate the occurrence of *P. profundisulcus* resting cysts in ship’s ballast tank arriving in the ports of China and North America, and (2) assess the potential bio-invasion risk of *P. profundisulcus* via ships’ ballast tank sediments.

## 2. Materials and Methods

### 2.1. Sampling and Sediment Collection

Ballast tank sediment sample CN was collected from an empty ballast tank with a capacity of 747.0 m^3^ from Vessel A arriving at Jiangyin Port, China. Vessel A is a general cargo ship built in 1997. The ballast water log of the studied tank before sampling was present in Table 1. The sediment sample was collected on 10 November 2017 with a sterile trowel and sub-packaged into sterilized tubes, which were stored at 4 °C in darkness until further analysis.

A ballast tank sediment sample NA was collected from a double-bottom tank of Vessel B entering Windsor Port (Ontario, Canada) on 26 June 2002. This sample was related to a research program for assessing potential invasion vectors via overseas vessels arriving in the Great Lakes, with the information of ship, tank, and ballasting history provided by Johengen et al. (2005) [31]. In detail, this investigated ship was built in 1986. During the research program, the voyage of this ship was from Tampico (Mexico) to Toledo (USA), Windsor (Canada), and Detroit (USA). During the voyage, it was ballasted at Everglades (USA) with a total ballast capacity of 9000 m^3^ in May 2002. The sampled tank was not cleaned at the dry dock, and the number of total residuals was about 50 metric tons with sediments <25% when sampling.

### 2.2. Culture of Pseudocochlodinium Profundisulcus Establishment from Ballast Sediment of Vessel A Collected from Jiangyin Port, China, Light and Scanning Microscopy

Ballast tank sediment sample CN from Vessel A (2 g) was used for the resting cyst germination experiment. Cyst assemblage was concentrated using sodium polytungstate solution [32]. Individual cysts were washed at least three times with sterile f/2 medium [33] and micropipetted to a 24-well culture plate with each well containing 2.5 mL fresh f/2 medium and one single cyst. An antibiotic solution (final concentration 2%; a mixture of 10,000 I.U. penicillin and 10,000 µg·mL^−1^ streptomycin, Solarbio, Beijing, China) and germanium dioxide (final concentration of 20 mg·L^−1^) was added into the medium immediately to discourage bacterial and diatom growth. The plate was incubated at 21 ± 1°C with 12:12 h light:dark cycle under a light intensity of 100 µmol photons m^−2^·s^−1^. A clonal culture of *P. profundisulcus* was established through germlings. The living cyst, empty cyst, and vegetative cells were observed with light microscopes (IX73, BX53, Olympus, Tokyo, Japan) and photographed by a DP80 digital camera (Olympus, Tokyo, Japan). Cells at exponential growth stage were fixed with osmium tetroxide (OsO_4_, 2% final concentration) for 40–50 min, gently filtered onto an 11 μm Millipore nylon membrane, dehydrated in an acetone series (10%, 30%, 50%, 70%, 90%, and 3 times in 100%, each step 15 min), critical point-dried (automated critical point dryer, EM CPD 300, Leica, Vienna, Austria), sputter-coated with gold (Sputter/Carbon Thread, EM ACE200, Leica, Austria), and observed with an S-3400N SEM (Hitachi, Hitachinaka, Japan).

### 2.3. Identification of Pseudocochlodinium Profundisulcus Cyst from North America

Ballast tank sediment sample NA collected from Vessel B entering North America was used to investigate the presence of *P. profundisulcus* cyst. Individual cysts were micropipetted from cyst assemblage concentrated using SPT from ballast sediment [32] and washed at least three times with sterile seawater enriched with f/2 medium [33]. A pair of primer (5′-ACCCGCTGAATTTAAGCATA-3′ and 5′-GCTACTACCACCAAGATCTGC-3′) was used for single-cell sequencing. The processes including PCR, cloning, and sequencing were according to Shang et al. (2019) [10].

### 2.4. DNA Extraction, PCR Amplification, Sequencing, and Phylogenetic Analyses

Total DNA of *P. profundisulcus* established from the Chinese port was extracted using a plant DNA extraction kit (Tiangen, Beijing, China) following the manufacturer’s protocol and identified by large subunit ribosomal DNA gene sequencing. About 1400 bp of LSU rDNA sequence was amplified using primers of D1R (forward, 5′-ACCCGCTGAATTTAAGCATA-3′) and 28-1483R (reverse, 5′-GCTACTACCACCAAGATCTGC-3′) [34,35]. The PCR reactions and sequencing were conducted according to Hu et al. (2021) [19].

Phylogenetic analyses for collected LSU rDNA sequences were aligned using the default settings of MAFFT v.7.110 [36,37] (Available online: http://mafft.cbrc.jp/alignment/server/; Accessed on 27 September 2021) and modified manually using BioEdit (v7.313) [38]. The program jModelTest 2.1.4 was executed to select the most appropriate model of molecular evolution with Akaike information criterion and GTR + G + I model with gamma-distributed rate variation across sites, and a proportion of invariable sites was chosen as the most appropriate model [39]. Maximum likelihood analysis was conducted with raxmlGUI v1.3.1 using the model GTR + I + G [40,41]. Node support was assessed with 1000 bootstrap replicates. The Bayesian inference analysis was performed by MrBayes 3.2.6 using the best-fitting substitution model (GTR + I + G) [42]. Four independent Markov chain Monte Carlo processes of 10,000,000 generations were conducted. Trees were sampled every 1000 generations. The first 10% of trees were discarded as burn-in. The convergence was assessed by the average standard deviation of split frequencies (less than 0.01). The remaining trees were used to generate a consensus tree, with posterior probabilities of all branches computed via a majority-rule consensus approach. FigTree (v1.4.4) was used to view and edit trees for publication.

## 3. Results

### 3.1. Morphology of Resting Cyst and Vegetative Cell of Pseudocochlodinium Profundisulcus

**Figure 1:**
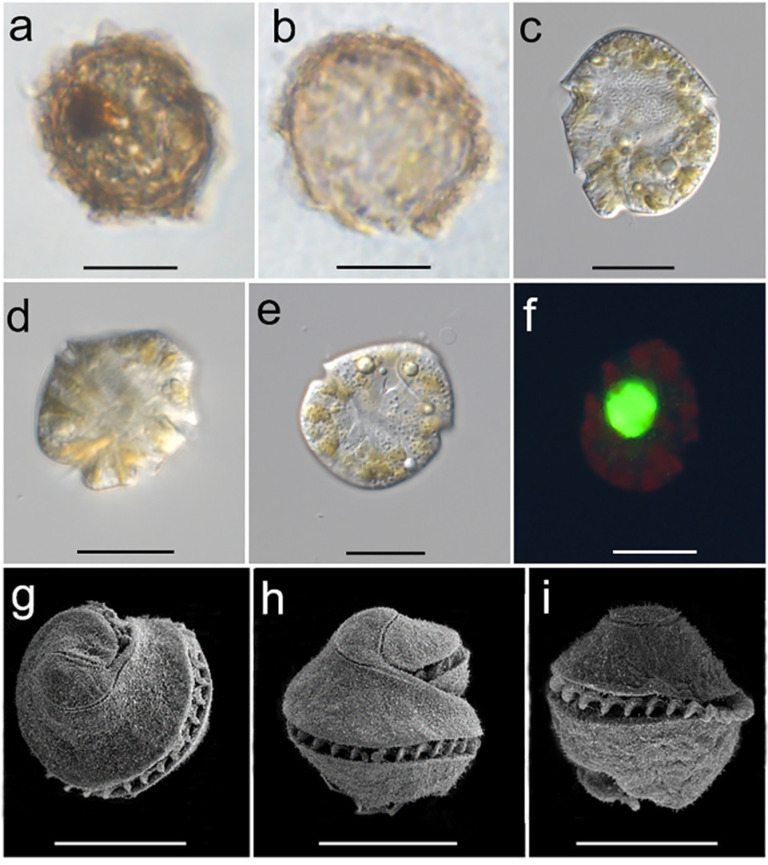
Light microscopic micrographs of the *Pseudocochlodinium profundisulcus* resting cyst (**a**), empty cyst (**b**), and vegetative cells (**c**–**f**); scanning electron microscopic micrographs (**g**–**i**) of the vegetative cells; Scale bars = 20 μm.

A resting cyst of *P. profundisulcus* in the Sample NA collected from a double-bottom tank of the Vessel B entering Windsor Port was isolated and observed, which was yellowish-brown, irregularly round with lobed ornaments around the cyst wall (identified as *Polykrikos geminatum*, see picture in Shang et al. (2019) [10]). In addition, two dark brownish resting cysts were isolated from the tank of Vessel A, arriving at Jiangyin port, China, and successfully germinated into vegetative cells in 7 days. Light and scanning electron microscopy were used to observe the characteristics of one of the resting cysts and the associated vegetative cells and provided the morphological structure for this species (Figure 1). The cysts of *P. profundisulcus* were about 45 μm in length and subspherical to spherical. The cyst wall was dark brownish in appearance with lobed ornaments (Figure 1a). A yellow to dark brownish accumulation body was present (Figure 1a). The archeopyle was chasmic (Figure 1b). Vegetative cells were roughly subspherical with a slightly conical convex epicone and round hypocone (Figure 1c–f). The nucleus was located near the center of the cell, and each cell contained many yellow-brownish to brown chloroplasts scattering throughout the cell periphery (Figure 1c–f). The cingulum encircled more than one, but less than one and a half turns of the cell body (Figure 1g–i). The apical structure complex was comma-shaped (Figure 1g–i).

### 3.2. Molecular Phylogeny

The accession numbers of LSU rDNA sequences for *P. profundisulcus* were obtained and deposited in NCBI (accession numbers: OK667806 (from China port) and OK667934 (from North America port)). The sequences were analyzed using the Basic Local Search Tool (BLAST, Available online: http://blast.ncbi.nlm.nih.gov/Blast.cgi; Accessed on 25 October 2021) in GenBank. The LSU rDNA sequences (OK667806 and OK667934) were found to be 98.5% and 97.54%, 99.8–99.9% and 99.2–99.5%, and 99.6–99.9% and 98.0%–99.5% identical to three entities in GenBank that were annotated as *Cochlodinium* cf. *geminatum* (EF616462), *Po. geminatus* (KF878934 and JX967270), and *P. profundisulcus* (MF445291, MG874046, MG874047, MG874048, MW811439, and MW811440)

**Figure 2:**
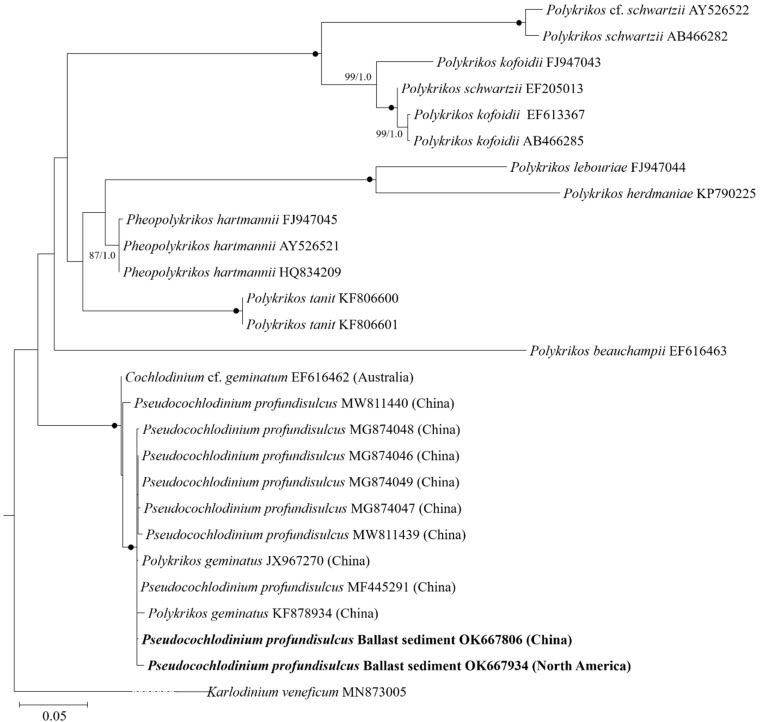
Phylogenetic tree of the *Pseudocochlodinium profundisulcus* based on the large subunit rDNA sequences. *Karlodinum veneficum* was used as an outgroup. Bootstrap values >50% and posterior probabilities (PP) above 0.5 are shown. Black dots (•) indicate maximal support (PP = 1.00 in BI and bootstrap support = 100% in ML, respectively).

Phylogenetic analyses using maximum likelihood and Bayesian inference generated similar trees but differed at a few internal nodes (Figure 2). The two entities (OK667806 and OK667934) from ballast sediments formed a coherent clade together with *P. profundisulcus* (MF445291, MW811439, MW811440, MG874046, MG874047, MG874048, and MG874049, from the Pearl River Estuary, China), a previously deposited entity of *Po. geminatus* (JX967270, KF878934, from the Pearl River Estuary, China; species name provided in GenBank), and *Cochlodinium* cf. *geminatum* (EF616462, from Australia; species name provided in GenBank) with strong support (100/1.0).

## 4. Discussion

Harmful algal blooms (HABs) are one of the most important marine ecological disasters. In the past few decades, algal blooms have occurred frequently in the world with an increasing trend [43,44,45,46]. Moreover, some HAB-forming species show a trend of spreading to other countries and regions [47,48,49]. In addition to global warming and water eutrophication associated with human activities, phytoplankton spread by ship’s ballast water and sediments is widely regarded as a very important external cause for the increase in HABs [1,49,50,51,52,53,54,55]. As one of the most ecologically important phytoplankton groups, dinoflagellates cause about 75% of the marine HABs events, and many species could produce various toxins [56]. In addition, dinoflagellates are important primary producers in the oceans, especially offshore, and molecular sequencing (e.g., rDNA) results showed that they accounted for about 50% of surface marine protists [57]. A very important biological feature of dinoflagellate is that many dinoflagellates, including these algal bloom species, can form dormant cysts during their life history [13]. The cysts could withstand harsh environment due to the thick cyst walls, allowing them to survive in marine sediments for a long period of time (from a few months to over 100 years) and germinate under suitable environmental conditions that provide seeds for reproduction and even forming blooms [12,13,14,15,16]. This characteristic of cysts has a very important ecological significance that makes them easier to be geographically dispersed through man-made or natural processes, such as the alien introduction by ballast water/sediments and farmed shellfish [1,50,54,55]. Smayda (2007) documented that those species clearly believed to be spread through ship’s ballast tanks to form harmful algal blooms in different places were all cyst- or dormant cell-producing species [54]. In turn, we inferred that the dinoflagellates that produce dormant cysts could, in theory, undergo allochthonous dispersal through the ballast tanks [58]. Since International Maritime Organization (IMO) established and adopted “The International Convention for The Control and Management of Ships Ballast Water Ballast Water and Sediments, 2004” (BWM Convention) at a Diplomatic Conference in London in 2004 and entered into force on 8 September 2017 [59], it is urgent to study the survivability of dinoflagellate cysts in an extreme environment for determining appropriate treatments on ballast sediments.

While the frequency, scale, and degree of harmful algal blooms have increased in China, it is particularly worth noting that new toxic and HABs-forming species have also been continuously uncovered [49]. As an organism that prefers high salinities and has a wide tolerance to temperature [23], *P. profundisulcus* was initially described as *Cochlodinium geminatum* Schütt [60] and transferred to the genus *Polykrikos* as *P. geminatus* by Qiu et al. (2013) [61]. Since the first record of *P. profundisulcus* in China in 2006, it has formed HABs frequently in the South China Sea since then [20,21,22,23,24,25]. Until now, *Pseudocochlodinium* species have had a global distribution [19]. In a previous study, we found 11 OTUs with 935 reads annotated as *Cochlodinium* sp., *Cochlodinium geminatum*, or *Polykrikos geminatus* (reclassified as *P. profundisulcus* by Hu et al. (2021) [19]) via metabarcoding approach, which implied many *P. profundisulcus* cysts present in the sediments of the ship’s tanks [10]. In this study, we further confirmed the presence of live resting cysts of *P. profundisulcus* in sediments of ballast tanks in an international vessel that had visited Brazil, USA, Japan, Australia, and China. As this ship loaded and discharged water in many places as well as in Huangdao Port, China, before our sampling, it was difficult to distinguish whether the cysts were from China or abroad. Moreover, we also identified a *P. profundisulcus* cyst by single-cyst polymerase chain reaction (PCR), and subsequent sequencing in the ballast tank sediment of an overseas vessel visited Mexico, USA, and Canada, which further indicated that this species could be transported as resting cyst via ship. Since *P. profundisulcus* has been reported from North America [62], the origin of the cyst in this ship is also a perplexing question.

The characteristic of *P. profundisulcus* to produce resting cysts could not only help to survive under unfavorable environmental conditions but also capable of enduring long-distance transport (currents, ballast tanks, or transplanted shellfish), which possibly facilitate its ability to colonize and settle in new habitats [2,10,17,54,58]. The initial outbreak and reoccurrence of *P. profundisulcus* bloom in China were observed near the harbor area where was subject to ship ballast waters [20,63]. In this study, we detected the *P. profundisulcus* resting cysts in the ship’s ballast tank sediments arriving in the ports of both China and North America. Moreover, we have germinated two *P. profundisulcus* cysts from one sediment sample (2 g) in the tank of Vessel A. This is important evidence that this toxic and harmful algal bloom-forming species not only exists as resting cysts in ships but also has the ability to reproduce, posing a serious threat to the accepted waters. Since dinoflagellates have the ability to reproduce sexually and form resting cysts, genetic exchange and recombination could happen in a short timescale [64,65,66]. Once the cysts in ballast tanks are de-ballasted into the accepted waters, they may hatch continuously in suitable growth conditions and supply novel genotypes to the indigenous ecosystems [67,68]. It is worthwhile mentioning that intraspecific genetical diversity is usually accompanied by substantial intraspecific trait variation, which can help the species to enhance its competitive success and adapt more quickly to changing environmental conditions [69]. As molecular tools offer the opportunity to identify genetic diversity, new insights into the physiology and ecology of phytoplankton are provided. For instance, distinct subpopulations succession of diatom (e.g., *Ditylum brightwellii*) and dinoflagellates (e.g., *Alexandrium catenella* and *Margalefidinium polykrikoides*) occurred to maintain long-term algal blooms [70,71,72]. This intraspecific variation ultimately results in the diversification and evolutionary adaptation of species, which can even happen over ecological time scales [73,74]. The high sequence homology found among the LSU rDNA sequences of *P. profundisulcus* in ballast tank sediments and the populations from China, Australia, and Japan deposited in the NCBI database, likely suggesting a recent common ancestor or origin for these *P. profundisulcus*. This stimulated us to propose a further investigation on the origination and genetic diversity of *P. profundisulcus* once there are sufficient molecular data from deep sediments and different regions. The presence of *P. profundisulcus* cysts in ship’s ballast tanks increased the potential of bio-invasion risk to China and North America since the dinoflagellate in tanks survived as resistant resting cysts during the voyage and hatched in the accepted ecosystems. This study suggests that corresponding supervision and treatment measures should be taken to deal with the invasion of alien species via ship’s ballast water and sediments.

## 5. Conclusions

Although the dinoflagellate *P. profundisulcus* forms blooms almost every year since 2006 in China, whether it is an indigenous or an invasive species remains unclear. In this study, we confirmed the presence of *P. profundisulcus* resting cysts in the ballast sediments from vessels arriving in the ports of China and North America. Moreover, two *P. profundisulcus* cysts were germinated into vegetative cells in the ballast sediments from Vessel A, which is a general cargo ship built in 1997 and docked numerous ports in Brazil, USA, Japan, and Australia, before sailing to Huangdao Port, Shandong Province, and Jiangyin Port, Jiangsu Province in China. As *P. profundisulcus* has been reported in many regions of the world, our results suggested that this organism might be extensively transferred via ships’ ballast tank sediments. The origin and genetic diversity of *P. profundisulcus* should be further explored to gather sufficient molecular data from different sources, including that from deep sediments and different countries.

## Figures and Tables

**Table 1:** The ballast water log for the studied ballast tank of Vessel A.

Start Point			End Point			Ballast Water Operation	**Remarks**
Date/Time	Location	Volume (m^3^)	Date/Time	Location	Volume (m^3^)		
31 May 2016/06:45	Rio Grande (Brazil)	1.20	31 May 2016/07:30	Rio Grande (Brazil)	700	Load	
4 June 2016/20:50	Rio Grande (Brazil)	700	4 June 2016/22:20	Rio Grande (Brazil)	1.20	Discharge	
2 August 2016/19:21	Savannah (USA)	1.20	2 August 2016/20:09	Savannah (USA)	635	Load	
10 August 2016/07:00	Port Arthur (USA)	635	10 August 2016/08:10	Port Arthur (USA)	0.7	Discharge	
16 September 2016/09:00	Sao Sebastiao (Brazil)	0.7	16 September 2016/09:50	Sao Sebastiao (Brazil)	634	Load	
19 September 2016/07:50	Paranagua (Brazil)	634	19 September 2016/08:55	Paranagua (Brazil)	0.9	Discharge	
13 November 2016/09:05	36°36.4′ N/122°32.1′ E	0.9	13 November 2016/09:57	36°43.2′ N/122°42.4′ E	635	Load	
3 December 2016/18:57	12°23.6′ N/129°14.0′ E	635	3 December 2016/19:46	12°15.5′ N/129°19.8′ E	2.1	Discharge	Empty
4 December 2016/08:01	10°26.0′ N/130°46.9′ E	2.1	4 December 2016/08:46	10°18.9′ N/130°51.7′ E	672.8	Load	Refill
16 January 2017/13:10	Gladstone (USA)	672.8	16 January 2017/14:00	Gladstone (USA)	0.6	Discharge	
20 February 2017/13:10	Yokohama (Japan)	0.6	20 February 2017/13:48	Yokohama (Japan)	573.2	Load	
23 February 2017/15:05	24°35.2′ N/140°40.1′ E	573.2	23 February 2017/16:15	24°20.9′ N/140°40.0′ E	1.20	Discharge	Empty
20 February 2017/16:18	24°20.4′ N/140°40.0′ E	1.20	20 February 2017/16:58	24°12.9′ N/140°40.2′ E	607.8	Load	Refill
23 March 2017/14:05	Newcastle (Australia)	607.8	23 March 2017/15:30	Newcastle (Australia)	0.9	Discharge	
20 August 2017/13:00	Yokohama (Japan)	0.9	20 August 2017/13:43	Yokohama (Japan)	635	Load	
29 August 2017/14:05	20°06.6′ N/130°46.5′ E	635	29 August 2017/15:30	19°51.2′ N/130°55.9′ E	0.9	Discharge	BWX ^1^
29 August 2017/17:00	19°34.8′ N/131°06.9′ E	0.9	29 August 2017/17:42	19°26.5′ N/131°12.4′ E	636	Load	BWX
18 September 2017/18:55	Portland (USA)	636	18 September 2017/20:15	Portland (USA)	0.7	Discharge	
28 October 2017/17:30	Huangdao (China)	0.7	28 October 2017/17:50	Huangdao (China)	293	Load	

^1^ BWX = Ballast water exchange.
